# Inverse dose-dependent effects of aripiprazole on sexual dysfunction and prolactin levels in patients with schizophrenia

**DOI:** 10.1192/j.eurpsy.2025.2230

**Published:** 2025-08-26

**Authors:** M.-L. Lu, K. K. Goh

**Affiliations:** 1 Department of Psychiatry, Taipei Medical University-Wan Fang Hospital, Taipei, Taiwan, Province of China

## Abstract

**Introduction:**

Aripiprazole, a novel antipsychotic drug with partial agonist activity at dopamine D_2_ and serotonin 5-HT_1A_ receptors, as well as antagonist activity at 5-HT_2A_ receptor, has demonstrated fewer side effects, including extrapyramidal symptoms, hyperprolactinemia, and metabolic disturbances, compared to other antipsychotic drugs.

**Objectives:**

The aim of this study was to examine the dose-dependent impact of aripiprazole on sexual dysfunction and prolactin levels in patients with schizophrenia. Patients with schizophrenia under aripiprazole monotherapy were recruited into this study.

**Methods:**

Patients with schizophrenia under aripiprazole monotherapy were recruited into this study. Psychopathology and sexual dysfunction were evaluated using the Positive and Negative Syndrome Scale and the Arizona Sexual Experiences Scale (ASEX), respectively. Fasting blood samples were analyzed to measure metabolic parameters and prolactin levels.

**Results:**

We recruited 128 patients with schizophrenia, comprising 86 females and 42 males. The prevalence of hyperprolactinemia, hypoprolactinemia, sexual dysfunction, and metabolic syndrome was 12.5%, 47.6%, 39.1%, and 36.7%, respectively. Patients with sexual dysfunction exhibited higher prolactin levels and a higher prevalence of hyperprolactinemia compared to those without sexual dysfunction. The ASEX scores showed a positive correlation with prolactin levels and a negative correlation with aripiprazole dose. Multivariate analysis revealed that prolactin levels and age were associated with ASEX scores. Prolactin levels were negatively correlated with aripiprazole dose and metabolic parameters. Multivariate regression analysis indicated that aripiprazole dose was associated with prolactin levels.

**Conclusions:**

Aripiprazole exhibited inverse dose-dependent effects on prolactin levels and ASEX scores in patients with schizophrenia. Regular monitoring of prolactin levels and sexual function is recommended for patients with schizophrenia receiving aripiprazole treatment.

**Disclosure of Interest:**

None Declared

